# Prevalence and Molecular Characterization of Methicillin-Resistant Staphylococci (MRS) and Mammaliicocci (MRM) in Dromedary Camels from Algeria: First Detection of SCC*mec*-*mecC* Hybrid in Methicillin-Resistant *Mammaliicoccus lentus*

**DOI:** 10.3390/antibiotics12040674

**Published:** 2023-03-29

**Authors:** Chahrazed Belhout, Filip Boyen, Nick Vereecke, Sebastiaan Theuns, Nadia Taibi, Marc Stegger, Pedro Yoelvys de la Fé-Rodríguez, Leila Bouayad, Rachid Elgroud, Patrick Butaye

**Affiliations:** 1 HASAQ Laboratory, High National Veterinary School, Issad Abbes Avenue, Oued Smar, El Harrach, Algiers 16270, Algeria; 2 Department of Pathobiology, Pharmacology and Zoological Medicine, Faculty of Veterinary Medicine, Ghent University, Salisburylaan 133, 9820 Merelbeke, Belgium; 3 PathoSense, Pastoriestraat 10, 2500 Lier, Belgium; 4 Department of Translational Physiology, Infectiology and Public Health, Faculty of Veterinary Medicine, Ghent University, Salisburylaan 133, 9820 Merelbeke, Belgium; 5 Centre de Recherche Scientifique et Technique en Analyses Physico-Chimiques (CRAPC), Bou-Ismail, Tipaza 42415, Algeria; 6 Department of Bacteria, Parasites & Fungi, Statens Serum Institut, Artillerivej 5, 2300 Copenhagen, Denmark; 7 Departamento de Medicina Veterinaria y Zootecnia, Facultad de Ciencias Agropecuarias, Universidad Central “Marta Abreu” de Las Villas, Carretera a Camajuaní km 5½, Santa Clara 54 830, Cuba; 8 Institute of Veterinary Sciences, University Frères Mentouri Constantine 1, Constantine 25017, Algeria

**Keywords:** dromedary camels, MRS, MRM, MRSA, M. lentus, SCC*mec*-*mecC*

## Abstract

Dromedary camels are an important source of food and income in many countries. However, it has been largely overlooked that they can also transmit antibiotic-resistant bacteria. The aim of this study was to identify the *Staphylococcaceae* bacteria composition of the nasal flora in dromedary camels and evaluate the presence of methicillin-resistant *Mammaliicoccus* (MRM) and methicillin-resistant *Staphylococcus* (MRS) in dromedary camels in Algeria. Nasal swabs were collected from 46 camels from seven farms located in two different regions of Algeria (M’sila and Ouargla). We used non-selective media to determine the nasal flora, and antibiotic-supplemented media to isolate MRS and MRM. The staphylococcal isolates were identified using an Autoflex Biotyper Mass Spectrometer (MALDI-TOF MS). The *mecA* and *mecC* genes were detected by PCR. Methicillin-resistant strains were further analysed by long-read whole genome sequencing (WGS). Thirteen known *Staphylococcus* and *Mammaliicoccus* species were identified in the nasal flora, of which half (49.2%) were coagulase-positive staphylococci. The results showed that four out of seven farms were positive for MRS and/or MRM, with a total of 16 isolates from 13 dromedary camels. The predominant species were *M. lentus*, *S. epidermidis*, and *S. aureus*. Three methicillin-resistant *S. aureus* (MRSA) were found to be ST6 and *spa* type t304. Among methicillin-resistant *S. epidermidis* (MRSE), ST61 was the predominant ST identified. Phylogenetic analysis showed clonal relatedness among *M. lentus* strains, while *S. epidermidis* strains were not closely related. Resistance genes were detected, including *mecA*, *mecC*, *ermB*, *tet*(K), and *blaZ*. An SCC*mec* type VIII element was found in a methicillin-resistant *S. hominis* (MRSH) belonging to the ST1 strain. An SCC*mec*-*mecC* hybrid element was detected in *M. lentus*, similar to what was previously detected in *M. sciuri*. This study highlights that dromedary camels may be a reservoir for MRS and MRM, and that they contain a specific set of SCC*mec* elements. This emphasizes the need for further research in this ecological niche from a One Health perspective.

## 1. Introduction

Dromedaries (*Camelus dromedarius*) are selectively bred, raised, and grazed for their milk and meat [1,2], which are used for human consumption. These camels are commonly found in arid areas and are almost exclusively domesticated for use as draught animals and food production [3]. The dromedary camel is common in Africa, the Middle East, Asia, and Australia [4]. In many semi-arid and arid regions of Africa, keeping dromedary camels is the most sustainable livestock enterprise [5]. Due to climate change and desertification, the numbers of cattle and small ruminants are decreasing in these regions, while camels are increasing in number. In the future, camels may become more important for people’s ability to earn a living or support themselves [6].

Camels were thought not to be affected by most of the diseases commonly impacting livestock [7]. However, recent data have confirmed their susceptibility to a high number of zoonotic pathogens [8]. Many zoonotic infectious diseases with their origin in dromedary have been an issue in Africa and the Middle East over the last decades, such as Middle East respiratory syndrome (MERS) and brucellosis [9,10]. Additionally, novel dromedary camel-borne diseases continue to be identified, as best exemplified by reports of a prion disease from Algerian abattoir camels [11]. Limited resources, low levels of regulation, poor hygiene, high mobility of camels and herders, and lack of consistent veterinary care also act as drivers for disease spill-over [12]. As more camels are raised for food production and climate change drives them into new territories, experts believe that the risk of zoonotic diseases transmitted from camels to humans is likely to increase [13]. The devastating effects of climate change on the agricultural and livestock industry have necessitated the need for extensive studies on camels as one of the animals most resistant to harsh environmental conditions [13,14]. It is a species that in the not-too-distant future could become a main source of food for large parts of Africa and Asia.

The family of the Staphylococcaceae currently comprises ten genera, including Abyssicoccus, Aliicoccus, Auricoccus, Corticicoccus, Jeotgalicoccus, Macrococcus, Mammaliicoccus, Nosocomiicoccus, Salinicoccus, and Staphylococcus [15]. Very recently, five well described staphylococcal species belonging to Staphylococcus sciuri group (S. sciuri, S. fleurettii, S. lentus, S. stepanovicii and S. vitulinus) were reassigned to the novel genus Mammaliicoccus, with Mammaliicoccus sciuri as the species type [15]. Staphylococci and mammaliicocci are considered to be opportunistic pathogens, but also being able to cause a wide range of severe infections [16,17]. They are notable for their ability to colonize a wide range of vertebrate hosts, with each host representing a distinct ecological niche [18]. Members of the M. sciuri group are often detected in animals, humans and the environment and generally considered harmless, but sometimes found in diseased animals [19]. M. sciuri has been isolated for example from bovine mastitis cases, from sick goats, piglets, and even from cases of canine dermatitis [17].

Little is known about the clinical importance of methicillin-resistant non-*aureus Staphylococcus* (MRNaS) and MRM [16,20]. However, these MRNaS/MRM can be carriers of antimicrobial resistance genes, and potentially able to act as donors of genes for other staphylococci species including *S. aureus* [19,21]. Resistance to β-lactams antibiotics in *Mammaliicoccus* and *Staphylococcus* is mediated by the production of either an alternative penicillin-binding protein with low affinity for β-lactams or a β-lactamase [22]. The β-lactamase, which is encoded by a *blaZ* gene, hydrolyses only penicillin-rings, hence conferring narrow-spectrum β-lactam resistance [23]. The alternative penicillin-binding protein is a transpeptidase encoded by the *mec* gene [24]. To date, four *mec* genes are known (*mecA, mecB, mecC,* and *mecD*). The *mecA* and *mecC* genes can be acquired by horizontal gene transfer through staphylococcal cassette chromosome *mec* (SCC*mec*) elements in *Staphylococcus* and *Mammaliicoccus*. The *mecB* and *mecD* genes have been found in *Macrococcus* on SCC*mec* elements, as well as on unrelated *mecD*-carrying *Macrococcus* resistance islands (McRI*_mecD_*) and large *mecB*-carrying plasmids [22], and only a single case of a plasmid borne *mecB*-positive staphylococci (*S. aureus*) has been reported [25]. Currently, 15 SCC*mec* types have been described in MRSA [26]. The distribution and complexity of SCC*mec* structures in *Staphylococcus* became more apparent with the extensive use of WGS and non-*aureus* staphylococci and mammaliicocci have been suggested as possible reservoirs of SCC*mec* elements [27]. The SCC*mec* element is a 20–70 kb genetic element inserted at a specific site (attachment site*, attB*) in *orfX* (a.k.a. *rmlH*, encoding 23S rRNA methyltransferase) near the replication origin of the chromosome [28,29]. Since 2011, *mecC* has been identified as a second determinant for methicillin resistance in *Staphylococcus* species. It was described in MRSA from humans [30,31] and dairy cattle [30] in Denmark, England, and the Republic of Ireland, but with an origin dating back centuries [32] and only reported from the European continent and New Zealand as well as in a single animal case from Australia [33]. Sharing 68.7% nucleotide with the archetypal *mecA* of *S. aureus* N315, this variant was designated as *mecC* and is encoded in MRSA within a distinct SCC*mec* classified as type XI [34].

In Africa, though less well known, antimicrobial resistance (AMR) is a growing concern in humans and animals [35]. The situation of both MRS and MRM in animals in these countries is not very well known, apart from some point prevalence studies and specific outbreaks [35,36]. However, recent research suggests that the epidemiology of MRS/MRM in Africa may be different from the other parts of the world due to the unique presence of *mecC* in staphylococci from animals and food [37]. Despite the fact that camels are an ecologically significant species in Africa, they have not been well studied and there is limited information on AMR in bacteria from camels [14]. The nasal carriage of staphylococci and mammaliicocci is not well studied, and there is even less information available on MRNaS and MRM. The human health risks associated with nasal colonization by these bacteria include food poisoning, skin infections, mastitis in dairy cows, and contaminated milk [37]. To our knowledge, there is only one study from Kenya that has investigated the presence of different *Staphylococcaceae* in camels and their antimicrobial resistance and virulence [38]. In Algeria, the numbers of dromedary camels have continuously increased in recent years. As these animals are kept in close contact to humans, they could be considered as source of human infections with MRS and MRM or as sources of mobile genetic elements carrying resistance genes that could be acquired be acquired by opportunistic bacteria more prone to colonization. Therefore, the aim of this study was to investigate the presence of different staphylococci and mammaliicocci strains in dromedary camels’ nostrils and to determine the prevalence and characteristics of MRS and MRM in healthy camels.

## 2. Results

### 2.1. Determination of Staphylococcaceae Flora in the Nose of Dromedary Camels

In this study, one hundred and twenty strains were isolated from forty-six healthy dromedaries in seven different farms in Algeria (Table 1). Thirteen known *Staphylococcus* and *Mammaliicoccus* species were identified. The results showed that coagulase-positive staphylococci (CPS) had the highest prevalence rate, accounting for 49.2% of the identified strains, followed by coagulase negative staphylococci (CNS) at 25.8%, while mammaliicocci accounted for 25.0% of the total strains.

Among the 59 CPS, *S. aureus* was the most commonly detected, with isolation rates of 54.2%, followed by *S. delphini* at 30.5% and *S. intermedius* at 15.3%. *S. aureus* and *S. delphini* were found in all seven farms. *M. sciuri* was the only *Mammaliicoccus* species detected, with a prevalence of 25.0%, and it was detected on six farms, with the highest occurrence on farm 1 (n = 10), followed by farms 2 and 3 (Table 1).

The nine different species of CNS species were distributed across the seven farms, with a prevalence ranging from 0.8% to 6.7%.

### 2.2. Detection of MRS and MRM in Dromedary Camels

Out of seven farms tested, four were positive for MRS and/or MRM, representing a proportion of 57%. Among these farms, one was located in the centre of Algeria (farm 1), while the remaining three were in the south of Algeria (farm 4, 5 and 6). On both farm 1 and farm 5, six out of ten dromedaries tested positive for MRS and/or MRM, resulting in a proportion of positive animals on these farms of 60%.

Sixteen MRS and MRM were recovered from thirteen different dromedaries distributed over the four positive farms. The identified species were *M. lentus* (n = 6), *S. epidermidis* (n = 6) *S. aureus* (n = 3), and *S. hominis* (n = 1).

### 2.3. WGS Analysis

#### 2.3.1. WGS and Phylogenetic Analysis

First, all assembled genomes from MRS/MRM isolates (n = 16) were quality checked using CheckM and pubMLST to assess genome completeness and perform species identification, respectively. The results of the MALDI-TOF MS classification and WGS-based classification using ribosomal genes (i.e., rMLST) was in concordance with each other. An overview of the species identification of both methods can be found in Supplementary File S1. The three MRSA isolates were found to be ST6 and *spa* type t304. Among methicillin-resistant *S. epidermidis* (MRSE), ST61 (n = 3) was the predominant ST. Other MRSE STs were ST54, ST640, and ST35, represented by one strain each. The MRSH was ST1. For the other species, no MLST scheme is available.

Analysis of the relatedness of the three isolated MRSA ST6/t304 using single nucleotide polymorphisms (SNPs) revealed that the strains were highly similar, with only two to five SNPs differentiating them. The *S. epidermidis* strains exhibited a greater degree of variation in this study and differed by 506 to 31,322 SNPs. Within ST61, the SNP difference was 506 to 546. As for *M. lentus,* five strains displayed clonal relatedness with a small range of SNP differences, from 9 to 47 SNPs, while one strain differed significantly, with over 4000 SNPs.

#### 2.3.2. Analysis of Antimicrobial Resistance Genes

Thirteen different antimicrobial resistance genes were identified by WGS in the 16 examined isolates (Table 2). All strains carried the methicillin resistance gene *mecA*, and in 5 *M. lentus mecC* was detected in addition to *mecA*. The other resistance genes present were responsible for resistance against penicillins (*blaZ*), aminoglycosides (*aadD*), trimethoprim (*dfrG*), lincosamides and macrolides (*erm(B), erm(A), msr(A), mph(C)*), fosfomycin (*fosB*), fuscidic acid (*fusB*, *fusC*), and tetracycline (*tet*(K)). The MRSA strains carried only genes responsible for resistance to β-lactams (*mecA, blaZ*), whereas MRNaS also carried other resistance genes. The *bleO* gene, responsible for bleomycin resistance, was detected only in MRSH.

**Figure 1:**
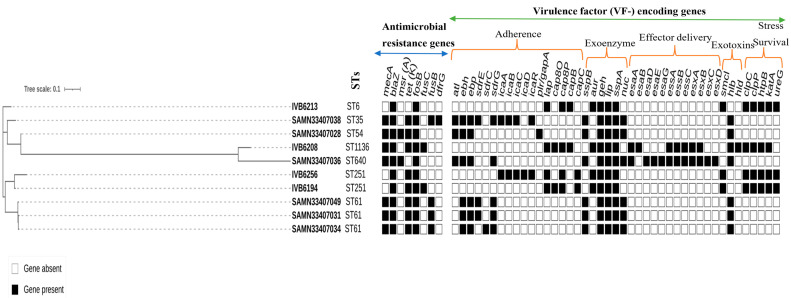
Phylogenetic analysis, antimicrobial resistance, and virulence encoding genes present in camel-derived S. epidermidis. The SNP-based phylogenetic tree was constructed with the strains of this study (SAMN33407028, SAMN33407031, SAMN33407034, SAMN33407036, SAMN33407038, SAMN33407049), and the strains of the previous study by Akarsu et al. (IVB6208, IVB6194, IVB6213, and IVB6256) [38]. Filled shapes indicate the presence of the genes. Empty shapes indicate absence of the genes. Scalebar indicates a genetic distance of 0.1 SNPs per branch.

To compare the distribution of AMR genes in *S. epidermidis* strains isolated from camels, the ten available strain sequences were examined (Figure 1). This included six strains from the current study (SAMN33407028, SAMN33407031, SAMN33407034, SAMN33407036, SAMN33407038, SAMN33407049) and four from a previous study by Akarsu et al. [38] (IVB6208, IVB6194, IVB6213, and IVB6256). Of the eight AMR genes detected, *blaZ* and *fosB* were present in all ten strains. Tetracycline resistance (*tet*(K)) was detected in eight isolates, with six strains from our study and two from Akarsu et al. [38]. Additionally, *mecA* was found in six strains from our study and one strain from the other study. The gene *fusc*, which mediates resistance to fuscidic acid, was detected in the isolates from the study of Akarsu et al. [38], whereas *fusB* was present in four (67%) strains from our study. Moreover, the *dfrG* gene was detected in only one strain among those isolated in this study (SAMN33407038).

#### 2.3.3. Analysis of Virulence Factors

A total of sixty-four virulence genes were found among the three MRSA ST6/t304 strains, while in MRSE twenty-eight virulence genes were identified. These genes encode factors for adherence, exoenzymes, immune evasions, exotoxins, effector delivery, and stress survival (Table 3). In the MRSA strains, a few examples of detected virulence genes are *hlgA*, *hlgB*, and *hlgC*, which encode the γ-hemolysin; *sak*, which encodes a staphylokinase; *scn,* which is associated with immune evasion; *aur*, which encodes aerolysin, the *sea* gene encoding enterotoxin; and the *lukC* and *lukD* genes, which encode a leukotoxin. The *icaABC* and its regulator *icaR,* which are responsible for biofilm formation, mucus production and its regulation, and facilitating attachment to environmental surfaces, were found in all MRSA strains and in one MRSE isolate (SAMN33407038).

The comparison of virulence genes in camel-derived *S. epidermidis* strains showed that the ten strains carried a total of forty-three virulence genes (Figure 1). Among the identified genes, some were exclusive to our strains: *atl*, *ebh*, *ebp*, *sdrE*, *sdrG, plr/gapA*, *esaD*, *esaE*, *esaG*, *esxC*, and *esxD.* Other genes were common to strains from both camel studies, including *icaA*, *icaB*, *icaR*, *geh*, *lip*, *sspA*, *nuc*, *esaA*, *essA*, *essB*, *essC*, *esxA*, *esxB*, and *hlb*.

#### 2.3.4. SCCmec Analysis

All three MRSA strains carried an SCC*mec* IVa element, while in the MRNaS strains, the SCC*mec* IIa was most often detected. This SCC*mec* IIa element was present in six strains, including four S. *epidermidis*, one *M. lentus*, and one *S. hominis.* Additionally, SCC*mec* IVa and SCC*mec* Vb were found in *S. epidermidis* (Table 2).

**Figure 2:**
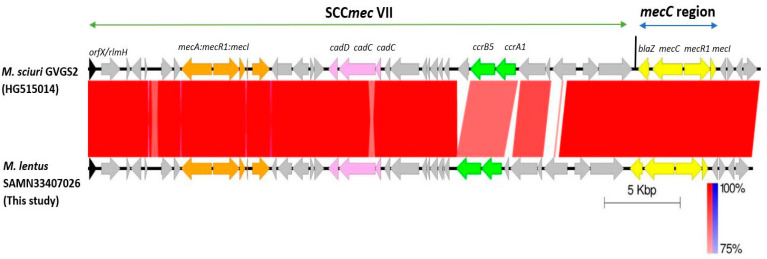
Schematic comparison of the SCCmec-mecC elements (SCCmec-mecC region) in M. lentus (SAMN33407026) and M. sciuri isolate GVGS2 (accession number HG515014) [39]. Selected key genes are highlighted and labelled. Regions of homology are represented by bands connecting the two sequences, with the percentage identity key shown on the bottom right. Red denotes normal sequence alignment and blue denotes inverted sequence alignment.

Genome sequencing of these five *mecA/C M. lentus* revealed that they all carried a SCC*mecA*-*mecC* hybrid element in the *orfX/rlmH* region, which was highly conserved between the isolates and with high similarity to that previously reported in *M. sciuri* GVGS2 (GenBank accession n^o^. HG515014) [39]. The SCC*mecA* was typed as SCC*mec* type VII and contains *mec* complex (*mecA*, *mecR1*, and *mecI*), a *cad* operon (*cad*ADC), encoding cadmium resistance and c*crA1*/*ccrB5* recombinases. The *mecC* region was part of a homologous class of E *mec* gene complex (*mecI-mecR1-mecC-blaZ*) (Figure 2).

## 3. Discussion

This is the first study specifically investigating MRS and MRM in dromedary camels. Because little is known on the nasal staphylococcal flora, we also included non-selective isolation of *Staphylococcus* and *Mammaliicoccus*. By non-selective isolation, we found 13 distinct species of *Staphylococcaceae*. Most of the strains were CPS (49.2%) with identification of *S. aureus*, *S. delphini*, and *S. intermedius*. The CPS group is commonly associated with severe infections [16]. Earlier studies conducted on healthy camels in several African and Middle Eastern nations have reported a variable prevalence of *S. aureus* in nasal carriage, ranging from 6.2% to 89.1% [40,41]. The reasons for this variability are unknown, though it may be due to differences in shared environments or urban lifestyles associated with breeding. Moreover, the presence of breeders or individuals in close contact with the animals may serve as potential sources of transmission.

Overall, these findings suggest that *S. aureus* is a common nasal colonizer of healthy camels with, however, significant variation in prevalence. The frequency of *S. aureus* isolated from camels in our study is similar to other studies conducted in Nigeria and higher than a recent study conducted in Tunisia [42,43]. Previous studies exploring the staphylococcal nasal flora of camels have reported the existence of different species, including *S. agnetis*, *S. arlettae*, *S. ureilyticus*, *S. delphini*, *S. felis*, *M. sciuri*, *M. lentus, S. muscae*-like, *S. pasteuri*, *S. chromogenes*, *S. schleiferi*, *S. epidermidis*, *S. hominis*, and *S. simulans* [38,42]. To our knowledge, this is the first study to identify *S. gallinarum*, *S. lutrae*, *S. intermedius*, *S. hyicus*, and *S. xylosus* in dromedary camels. Further studies are necessary to understand the staphylococcal flora of the nose of camels.

In the past decades, there has been a growing concern regarding the issue of the staphylococci of animals, especially related to methicillin resistance. Thorough investigations into MRS/MRM in camels are infrequent, particularly those that involve nasal carriage, nor have there been a lot of strains subjected to WGS. Our results indicate that more than half of the farms tested were positive for MRS/MRM, though there seem to be regional differences. We found a high diversity of species and clones, indicating that methicillin resistance is widespread. Similar findings has been reported from Kenya and Saudi Arabia [42,44]. The most common MRS and MRM species were *M. lentus* and *S. epidermidis*, which confirms the findings of Silva et al. (2022) [42], which also found *M. lentus* as the most prevalent methicillin-resistant species. These species are the most prevalent coagulase negative staphylococci at the clinical level and as part of the normal nasal microbiota of healthy individuals, which may suggest a possible human origin [45,46]. No susceptible *M. lentus* were found in either study. Further research is needed to fully understand the prevalence and mechanisms of methicillin resistance in *M. lentus*. Our results on the prevalence of MRS will also allow us to set up a surveillance with a more accurate sample size estimation.

The *mecA* carried on SCC*mec* elements is considered to originate from members of the *Mammaliicoccus* group (*M. sciuri*, *M. lentus*, *M. vitulinus*, and *M. fleurettii*), and the members of this group played a major role in the evolution and dissemination of the *mecA* gene [47]. In this study, five out of six *M. lentus* strains carried *mecA* and *mecC* genes; these strains were very similar and support a clonal spread of this resistant bacterium. The *mecC* was found encoded within a hybrid SCC*mec* element comprised of a *mecA* encoding SCC*mec* type VII and an *mecC* region. This element has been described in three *M. sciuri*, originally in GVGS2 from a caesarean incision wound in a Belgian Blue cow from England [39], LP600 from an alpaca in Austria [48], and in EC1S1 strains isolated from cattle and calves in Tunisia [49]. The *mecC*-carrying isolates were *M. sciuri* subsp. *carnaticus*, supporting the existence of a potential reservoir for *mecC*-carrying in *M. sciuri* [39,48,49]. In addition, the SCC*mec*-*mecC* element has been reported in a single *S. aureus* isolate from a racehorse wound [50]. In each case, the SCC*mecA*-*mecC* hybrid element was highly conserved, with the archetypal version reported originally in GVGS2 [39].

It is the first time this hybrid SCC*mec* has been identified in *M. lentus*, in dromedary camels, and in Algeria, while the molecular epidemiology of *M. lentus* in the farm environment was largely unknown. The origin of this element is yet to be uncovered. The *mecC* gene has been associated with wildlife, especially hedgehogs [51,52], and has been detected in many countries but at rather low prevalence, except for South African isolates [37]. Most countries also have hedgehogs among their wildlife, but those have not been studied in detail in most parts of the world. A study on the prevalence of MRS/MRM in wildlife in this region, with a focus on hedgehogs, may be needed to decipher the origins and spread of *mecC*.

Three MRSA strains identified as ST6/t304 were near identical, and all harboured an SCC*mec* Iva, which is commonly found in this ST [53]. This ST6/t304 is a typical community-acquired type (CA-MRSA) and is commonly found in Asia, as well as in northern Europe [54,55]. The MRSA ST6/t304 has also been found in broiler meat in Germany [56] and in pigs in China [57] and recently in humans and hedgehogs in Finland [58]. This raises concerns about the transmission of MRSA between humans and animals, which could have serious implications for public health. In addition, data from Finland and Denmark revealed that ST6/t304 was the most common ST found in MRSA isolates among refugees from the Middle East [59]. In Algeria, the MRSA-ST80 clone is the predominant clone found in nasal carriage, human samples, animals, food, and water [40,60]. The origin of the MRSA ST6/t304 in dromedary camels in Algeria remains thus unknown and needs further investigation from a One Health perspective.

In MRSE, ST61 was the most predominant ST identified in dromedaries with SCC*mec* IIa, which was the most predominant SCC*mec* element in these strains. MRSE ST61 has been found in association with infections in humans [61]. There are no data on the prevalence and STs of MRSE in Algeria. This lack of data makes it difficult to determine the origin of these strains in dromedary camels, though it raises the concern that these strains may be highly prevalent in the region. The MRSH strain isolated in this study belongs to ST1, which is the most prevalent ST of the *S. hominis* subsp. *hominis*. The strain carried the SCC*mec* VIII.

All MRNaS isolates analysed in this study had additional resistance genes, in contrast to the MRSA ST6/t304 strain that only had the *blaZ* and *mec* genes. It is worth noting that previous studies on MRSA ST6/t304 strains had identified various other genes, including *ermC*, *tet*(K), *fusC*, *lnuA*, and *aadA* [55]. Most strains (except for one, *M. lentus*) harboured the *blaZ* gene. These results are consistent with a previous study in Kenya where all the isolates from dromedary camels harboured the *blaZ* gene [38]. Apart from these β-lactamase encoding genes, the *tet*(K) gene was highly prevalent. This gene is commonly found in *Staphylococcus* and *Mammaliicoccus* species, including *S. epidermidis* [62]. The presence of the *tet*(K) gene can be a concern in African countries, where tetracycline antibiotics are commonly used for treatment due to their affordability and availability.

Interestingly, the bleomycin resistance gene (*bleO*) was detected in MRSH ST1 isolates. The bleomycin is commonly used for the treatment of various cancers, but it is not a commonly used antibiotic for bacterial infections [63]. Therefore, it is unlikely that the presence of the *bleO* gene is related to selective pressure from the use of bleomycin in the treatment of bacterial infections. Instead, it is more likely that the *bleO* gene was acquired through horizontal gene transfer from other bacteria in the environment that have been exposed to bleomycin [64]. This could have happened through the transfer of plasmids, transposons, or other mobile genetic elements that carry the resistance gene. This finding is significant because it highlights the potential for bacteria to develop resistance to drugs that are not traditionally used to treat bacterial infections [63].

Comparison of distribution of antimicrobial resistance genes among camel-derived *S. epidermidis* from different STs isolated in the current and a previous study by Akarsu et al. [38] showed that these isolates harboured different antimicrobial resistance genes in common, including *mecA*, *blaZ*, and *tet*(K). In both studies, the camels were in traditional farming communities, and the antibiotics may not be widely available or affordable, leading to limited or no use of antibiotics in treating bacterial infections, and thus a low selective pressure is assumed. However, there are few studies available on the general level of antimicrobial use in dromedaries or antimicrobial resistance in bacteria from dromedaries, and the interpretation remains difficult [40,41].

Different virulence-encoding genes have been detected in MRSA and MRSE strains with little variation. These virulence factors are associated with several key functions, including adherence, exoenzyme production, exotoxin production, effector delivery, and stress survival, all of which are important for pathogenicity. The result that MRSA ST6/t304 isolates were negative for Panton–Valentine leukocidin (PVL) is consistent with previous findings in northern Europe and Asia [54,55]. Similar results were found in the study of Chehida et al. [41]. Comparison of virulence factors among camel-derived *S. epidermidis* showed that the strains harboured different virulence genes that were not similar. The fact that the *S. epidermidis* strains isolated from both camel studies have different characteristics suggests that these strains may have adapted to the host and developed a distinct set of virulence factors.

The finding that dromedary camels in Algeria harbour MRS and MRM, as well as the hybrid element SCC*mec*-*mecC*, suggest that dromedaries may serve as a reservoir for antibiotic-resistant staphylococci, potentially leading to zoonotic transmission to humans. As such, this research highlights the importance of continued surveillance and monitoring of antibiotic resistance in both animal and human populations to mitigate the spread of antibiotic-resistant bacteria and preserve the effectiveness of antimicrobial therapies.

## 4. Materials and Methods

### 4.1. Sample Origin and Isolation Methods

**Figure 3:**
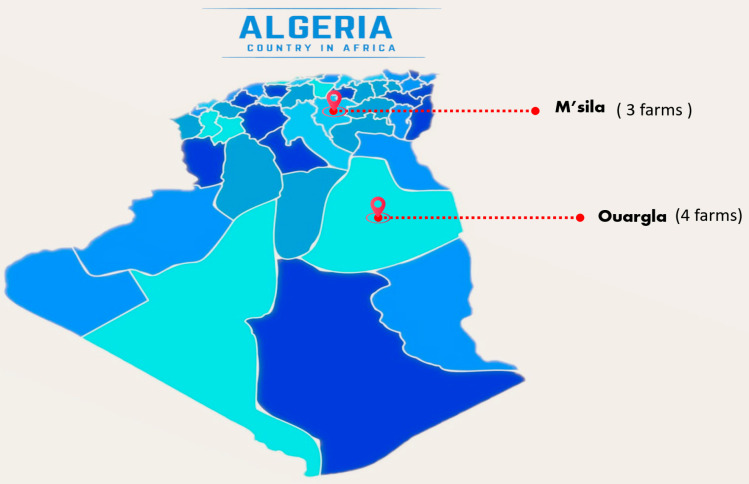
Sample collection sites. (In the centre of Algeria: M’sila, in the south of Algeria: Ouargla).

Between December 2021 and June 2022, a total of forty-six dromedaries (*Camelus dromedarius*) were sampled from seven Algerian farms present in two different regions (three farms located in the centre of Algeria (M*’*sila) and four farms in the south (Ouargla)) (Figure 3). We collected samples from these two different regions based on the higher density of camels and the accessibility of the regions. The number of dromedary camels selected for swabbing on each farm ranged from 5 to 10, and they were chosen at random (Supplementary Table S2). These dromedaries were observed to be healthy, and they had not received antibiotics at least three months before based on the information provided by the veterinarians and camel owners. Nasal swabs were transported in an icebox to the laboratory within 4 to 6 h for further analysis.

#### 4.1.1. Isolation of Non-Methicillin-Resistant *Staphylococcaceae*

Staphylococcaceae were first enriched in 6.5% NaCl Brain Heart Infusion (BHI) broth (HiMedia, Mumbai, India) at 37 °C overnight, and then sub-cultured onto Columbia agar (Oxoid, Hampshire, UK) supplemented with 5% sheep blood, after which the plates were incubated at 37 °C for 24–48 h. Colonies that resembled staphylococci or mammaliicocci were chosen based on their distinct morphologies, and purified on Columbia agar with 5% sheep blood. The isolates were grown at 37 °C for 24–48 h, and subsequently stored at −80 °C in 50% BHIB and 50% glycerol until further analysis.

#### 4.1.2. Isolation of Methicillin-Resistant *Staphylococcaceae*

The method used for isolating methicillin-resistant *Staphylococcaceae* involved some adjustments to the previously described protocol [65]. Firstly, each sample was cultured overnight at 37 °C in 6.5% NaCl BHI broth (HiMedia, Mumbai, India), after which it was sub-cultured on two different types of agars: Oxacillin Resistance Screening Agar Base (ORSAB) and Columbia Agar (Oxoid, Hampshire, UK) supplemented with 5% sheep blood and antibiotics (3.5 mg/L cefoxitin and 4 mg/L colistin). Following this, the plates were incubated at 37 °C for 24–48 h. Colonies with various morphologies resembling staphylococci or mammaliicocci were selected and purified on Columbia agar with 5% sheep blood and incubated for 24–48 h at 37 °C, and then stored at −80 °C in 50% BHIB and 50% glycerol until further analysis.

### 4.2. Bacterial Identification

The isolated bacteria were presumptively identified as *Staphylococcus* and *Mammaliicoccus* using the catalase test. Subsequent species identification was performed using the Autoflex Biotyper Mass Spectrometer (MALDI-TOF MS) (Bruker Daltonics, Bremen, Germany) via the direct transfer method with α-cyano-4-hydroxycinnamic acid as the matrix, following the manufacturer’s protocol. If no peaks were detected, the samples were retested using formic acid treatment. The resulting spectra were analysed using the MBT (MALDI BioTyper) compass software version 4.1. (Bruker Daltonics, Bremen, Germany) as previously described [66].

### 4.3. PCR for mec Genes Identification

One strain per species per animal was selected for PCR. The DNA was extracted as previously described [67] using lysostaphin and proteinase K. First, a simplex PCR targeting the *mecA* gene was performed as described previously [68]. Strains negative for *mecA* were subsequently tested for the presence of the *mecC* gene. The primers used are listed in Supplementary Table S3 [68]. The amplified DNA fragments were separated by 1.5% agarose gel electrophoresis (Sigma–Aldrich, St. Louis, MO, USA) stained with Midori Green Direct, for 1 h 15 min at 175V, using a Gene Ruler 100 bp DNA ladder.

### 4.4. Whole Genome Sequencing (WGS)

Sixteen mec-positive isolates were selected for WGS using Oxford Nanopore Sequencing (ONT), as previously described [69,70]. Colonies of a freshly grown culture were collected in 250 µL dPBS. High molecular weight (HMW) DNA was isolated using the ZymoBIOMICS DNA MiniPrep kit (Zymo Research, Irvine, CA, USA) at the PathoSense laboratory. Manufacturer’s instructions were followed, with the addition of a 30-min Proteinase K treatment (20 μg.µL^−1^; Promega, Madison, WI, USA). Bead bashing was performed with a TissueLyzer (Qiagen, Hilden, Germany) twice for 5 min at 30 oscillations per minute. The DNA quality was verified using a Nanodrop Spectrophotometer. If A260/A230 or A260/A280 were below 1.7, DNA was further cleaned using CleanNGS (CleanNA, Waddinxveen, The Netherlands) magnetic beads in a 1:1 ratio. High-quality HMW DNA was subjected to a rapid library preparation (SQK-RBK004; ONT) with 400ng DNA input per isolate. A maximum of 12 isolates were multiplexed on a single R.9.4.1 flow cell. Sequencing was carried out on a GridION device (ONT), allowing raw database calling and demutiplexing (guppy v6.3.9 with sup-accurate model; ONT) using the MinKNOW software (ONT). An overview of sequencing output and coverage can be found in Supplementary Table S1.

#### 4.4.1. Genome Assembly and Annotation

The fastq files were used in an in-house bacterial whole genome assembly pipeline using the Trycycler pipeline v0.5.3 [71]. In short, reads were filtered using filtlong v0.2.1 (--min_length 1000 --keep_percent 95; https://github.com/rrwick/Filtlong, accessed on 10 October 2022), prior to subsampling into 10 subsamples (--min_read_depth 50 --count 10 --genome_size 5M) [71]. Each subsample was used to perform an initial genome assembly using either flye v2.9 [72], raven v1.8.1 [73], miniasm_and_minipolish.sh v0.3 (https://github.com/rrwick/Minipolish, accessed on 10 October 2022), or wtdbg2 v1.12 [74], as instructed on the Trycycler manual page. *Default* settings were used to generate consensus genomes in Trycycler, including removal of contigs with coverage below 10% of the median sample sequencing coverage, clustering, reconciling, multiple sequence alignment, read partitioning, and generation of complete circular genome and plasmid consensuses. Final outputs were still subjected to read mapping and polishing using minimap2 v2.20 [75] and medaka v1.5.0 (ONT), respectively. The completeness and accuracy of final consensus genomes was assessed using ribosomal multi-locus sequence typing (rMLST) [76] and CheckM v1.1.0 [77]. A genome was considered complete based on 773 marker genes from 60 *Staphylococcus* genomes. When all marker genes were identified, a completeness of 100% was reported. The assembled contigs were annotated using the automated RAST server (Rapid Annotation using Subsystem Technology) [78].

#### 4.4.2. Core Genome Analysis

Multilocus sequence typing (MLST) was conducted using the MLST Finder v2.0.4, and *spa* typing was carried out with SpaTyper v1.0, both of which are available through the Centre for Genomic Epidemiology (https://cge.cbs.dtu.dk/services/, accessed on 10 October 2022). The CSI phylogeny 1.4 tool available on the Centre for Genomic Epidemiology (CGE) (https://cge.cbs.dtu.dk/services/CSIPhylogeny/, accessed on 10 October 2022) was used, with default parameters for phylogenetic analysis. The resulting phylogenetic trees were visualized using iTOL v6.3.2. [79].

#### 4.4.3. Accessory Genome Analysis

The assembled files were analysed for the presence of resistance genes using ResFinder v.3.0 (https://cge.cbs.dtu.dk/services/ResFinder/, accessed on 12 October 2022). Virulence factors were detected using VirulenceFinder 2.0 (https://cge.food.dtu.dk/services/VirulenceFinder/, accessed on 12 October 2022) and the virulence factor database (VFDB) [80]. The SCC*mec* typing scheme was used to assign MRS isolates to the appropriate subtype using the SCC*mec*Finder v1.2 online tool, available through the Centre for Genomic Epidemiology (https://cge.food.dtu.dk/services/SCCmecFinder/, accessed on 12 October 2022) and blast for the hybrid SCC*mec* and visualized using EasyFig.

## 5. Conclusions

In conclusion, the *Staphylococcaceae* nasal flora in dromedary was further characterized, and five species that were never reported before in dromedaries were found. Nearly half of the species detected were CPS, with *S. aureus* as the most frequent species. The prevalence of MRS/MRM at farm level was 57%, and *M. lentus* and *S. epidermidis* as the most frequent species. CA-MRSA was also detected, and the origin of these strains is probably human, indicating cross contamination between humans and dromedary camels. Similarly, most of the MRSE were ST61, typically found in human infections. The novel variant of the staphylococcal SCC*mec* type VIII element has been identified in MRSH. All but one of the *M. lentus* strains had the rare SCC*mec*-*mecC* hybrid element, indicating a specific epidemiology of this element in Algerian camel *Staphylococcaceae*. Moreover, this is the first time that this element was detected in *M. lentus*. These results are an indication for a specific epidemiology in Algeria, where *mec*C seems to be more associated with methicillin resistance than elsewhere in the world, though the low number of publications from that region needs further investigation. One Health-inspired research, including all compartments, on the prevalence and types of MRS and MRM are necessary to elucidate the epidemiology and spread of methicillin resistance, as this resistance compromises health by a large degree.

## Figures and Tables

**Table 1:** Distribution of different camel-derived *Staphylococcaceae* strains in the seven farms.

*Staphylococcaceae Species*	Number of Strains	Source
*S. aureus*	32	farm 1 (n = 9), farm 2 (n = 1), farm 3 (n = 8), farm 4 (n = 5), farm 5 (n = 2), farm 6 (n = 3), farm 7 (n = 4)
*M. sciuri*	30	farm 1 (n = 10), farm 2 (n = 3), farm 3 (n = 9), farm 5 (n = 3), farm 6 (n = 3), farm 7 (n = 2)
*S. delphini*	18	farm 1 (n = 1), farm 2 (n = 1), farm 3 (n = 8), farm 4 (n = 2), farm 5 (n = 1), farm 6 (n = 2), farm 7 (n = 3)
*S. intermedius*	9	farm 3 (n = 4), farm 5 (n = 2), farm 7 (n = 3)
*S. simulans*	7	farm 3 (n = 3), farm 4 (n = 3), farm 7 (n = 1)
*S. agnetis*	4	farm 1 (n = 1), farm 2 (n = 1), farm 3 (n = 1), farm 5 (n = 1)
*S. epidermidis*	2	Farm 3 (n = 1), farm 4 (n = 1)
*S. hyicus*	8	farm 1 (n = 2), farm 2 (n = 1), farm 6 (n = 3), farm 7 (n = 1)
*S. chromogenes*	4	farm 1 (n = 2), farm 3 (n = 1), farm 4 (n = 1)
*S. lutrae*	1	farm 5 (n = 1)
*S. gallinarum*	1	farm 5 (n = 1)
*S. muscae*	2	farm 3 (n = 2)
*S. xylosus*	2	farm 4 (n = 1), farm 7 (n = 1)

n: number of positive dromedaries in each farm.

**Table 2:** Distribution of detected antimicrobial resistance genes in methicillin-resistant *Staphylococcus* and *Mammaliicoccus*.

Biosamples Accessions Numbers	Species	Farm (Origin)	Region	Sequence Type ST *	SCC*mec*	β-Lactams	Other Antibiotic Resistance Genes
SAMN33407026	*M. lentus*	1	Centre (M’sila)	NA	SCC*mec* VII -*mecC*	*mecA, mecC, blaZ*	*str, erm(B), mph(C), tet*(K)
SAMN33407029	*M. lentus*	1	Centre (M’sila)	NA	SCC*mec* VII -*mecC*	*mecA, mecC, blaZ*	*str, erm(B), mph(C), tet*(K)
SAMN33407050	*M. lentus*	5	South (Ouargla)	NA	SCC*mec* VII -*mecC*	*mecA, mecC, blaZ*	*str, erm(B), mph(C), tet*(K)
SAMN33407025	*M. lentus*	1	Centre (M’sila)	NA	SCC*mec* VII -*mecC*	*mecA, mecC, blaZ*	*str, erm(B), mph(C)*
SAMN33407043	*M. lentus*	1	Centre (M’sila)	NA	SCC*mec* VII -*mecC*	*mecA, mecC, blaZ*	*erm(B), mph(C), tet*(K)
SAMN33407027	*M. lentus*	1	Centre (M’sila)	NA	SCC*mec* IIa	*mecA*	*erm(A), mph(C)*
SAMN33407030	*S. aureus*	4	South (Ouargla)	ST6	SCC*mec* IVa	*mecA, blaZ*	
SAMN33407032	*S. aureus*	4	South (Ouargla)	ST6	SCC*mec* IVa	*mecA, blaZ*	
SAMN33407033	*S. aureus*	5	South (Ouargla)	ST6	SCC*mec* IVa	*mecA, blaZ*	
SAMN33407028	*S. epidermidis*	1	Centre (M’sila)	ST54	SCC*mec* Vb	*mecA, blaZ*	*tet*(K), *fosB, msr(A)*
SAMN33407031	*S. epidermidis*	4	South (Ouargla)	ST61	SCC*mec* IIa	*mecA, blaZ*	*tet*(K), *fosB, fusB*
SAMN33407034	*S. epidermidis*	5	South (Ouargla)	ST61	SCC*mec* IIa	*mecA, blaZ*	*tet*(K), *fosB, fusB*
SAMN33407036	*S. epidermidis*	5	South (Ouargla)	ST640	SCC*mec* IVa	*mecA, blaZ*	*msr(A), fosB*
SAMN33407038	*S. epidermidis*	6	South (Ouargla)	ST35	SCC*mec* IIa	*mecA, blaZ*	*tet*(K), *fosB, fusB, dfrG*
SAMN33407049	*S. epidermidis*	5	South (Ouargla)	ST61	SCC*mec* IIa	*mecA, blaZ*	*tet*(K), *fosB, fusB*
SAMN33407035	*S. hominis*	5	South (Ouargla)	ST1	SCC*mec* VIII	*mecA, blaZ*	*bleO, aadD, tet*(K), *fusC*

* Only when a sequence typing scheme was available. NA: Not applicable.

**Table 3:** Distribution of detected virulence factors encoding genes in MRSA and MRSE.

Biosamples Accessions Numbers	Species	Adhesion	Exoenzymes	Immune Evasion	Effector Delivery	Exotoxins
SAMN33407030	*S. aureus*	*atl, ebh, cna, ebp, eap/map, efb, fnA, fnB, icaA, icaB, icaC, icaR, sdrC, sdrD, sdrE, spa,*	*sspB, sspC, hysA, geh, lip, sspA, coa, sak, nuc,*	*adsA, chp, scn, sbi*	*esaA, esaB, esaE, esaG, essA, essB, essC, esxA, esxB, esxC, esxD*	*hly/hla, hlb, hld, sea, hlgA, hlgB, hlgC, lukD*
SAMN33407032	*S. aureus*	*atl, ebh, cna, ebp, eap/map, efb, fnA, fnB, icaA, icaB, icaC, icaR, sdrC, sdrD, sdrE, spa*	*sspB, sspC, hysA, geh, lip, sspA, coa, sak, nuc*	*adsA, chp, scn, sbi*	*esaA, esaB, esaE, esaG, essA, essB, essC, esxA, esxB, esxC, esxD*	*hly/hla, hlb, hld, sea, hlgA, hlgB, hlgC, lukD*
SAMN33407033	*S. aureus*	*atl, ebh, cna, ebp, eap/map, efb, fnA, fnB, icaA, icaB, icaC, icaR, sdrC, sdrD, sdrE, spa*	*sspB, sspC, hysA, geh, lip, sspA, coa, sak, nuc*	*adsA, chp, scn, sbi*	*esaA, esaB, esaE, esaG, essA, essB, essC, esxA, esxB, esxC, esxD*	*hly/hla, hlb, hld, sea, hlgA, hlgB, hlgC, lukD*
SAMN33407028	*S. epidermidis*	*atl, ebh, ebp, plr/gapA*	*sspB, geh, lip, sspA, nuc*			*hlb*
SAMN33407031	*S. epidermidis*	*ebh, ebp, sdrE, sdrG,*	*sspB, geh, lip, sspA, nuc*			*hlb*
SAMN33407034	*S. epidermidis*	*ebh, ebp, sdrC, sdrG*	*sspB, geh, lip, sspA, nuc,*			*hlb*
SAMN33407036	*S. epidermidis*	*atl, ebh, ebp, sdrG*	*sspB, geh, lip, sspA, nuc*		*esaA, esaD, esaE, esaG, essA, essB, essC, esxA, esxB, esxC, esxD*	*hlb*
SAMN33407038	*S. epidermidis*	*atl, ebh, ebp, sdrE, sdrG, icaA, icaB, icaC, icaR,*	*sspB, geh, lip, sspA, nuc*			*hlb*
SAMN33407049	*S. epidermidis*	*ebh, ebp, sdrE, sdrG*	*sspB, geh, lip, sspA, nuc*			*hlb*

## Data Availability

The sequences and annotations of the chromosomes are available in the NCBI under project no. PRJNA937407 with the following accession numbers: SAMN33407026, SAMN33407029, SAMN33407050, SAMN33407025, SAMN33407043, SAMN33407027, SAMN33407030, SAMN33407032, SAMN33407033, SAMN33407028, SAMN33407031, SAMN33407034, SAMN33407036, SAMN33407038, SAMN33407049, SAMN33407035.

## Supplementary Materials

The following supporting information can be downloaded at: https://www.mdpi.com/article/10.3390/antibiotics12040674/s1, Table S1: Overview of *Staphylococcus* and *Mammaliicoccus* genomes; Table S2: Number of Dromedary Nasal Swab Samples Collected in Each Farm; Table S3: Sequence of primers used for *mec* gene amplification in this study.
